# KpSC-ID: a multiplex real-time PCR assay for the simultaneous detection of the Klebsiella pneumoniae species complex and specific identification of Klebsiella pneumoniae, Klebsiella quasipneumoniae and Klebsiella variicola

**DOI:** 10.1099/mic.0.001587

**Published:** 2025-07-30

**Authors:** Grainne McAndrew, Elodie Barbier, Carla Rodrigues, Pascal Piveteau, Sylvain Brisse, Kate Reddington

**Affiliations:** 1 Microbiology, School of Biological and Chemical Sciences, University of Galway, Galway, Ireland; 2 UMR Agroécologie, INRAE, AgroSup Dijon, Université de Bourgogne, Dijon, France; 3 Institut Pasteur, Université Paris Cité, Biodiversity and Epidemiology of Bacterial Pathogens, F- 75015, Paris, France; 4 INRAE, UR OPAALE, Rennes, France

**Keywords:** differentiation, identification, *Klebsiella pneumoniae* species complex

## Abstract

The *Klebsiella pneumoniae* species complex (KpSC) comprises five closely related bacterial species, namely *Klebsiella pneumoniae*, *Klebsiella quasipneumoniae*, *Klebsiella variicola*, *Klebsiella quasivariicola* and *Klebsiella africana*. The KpSC is ubiquitous in the environment and is also an important human pathogen, particularly associated with healthcare-associated infections. The accurate detection and differentiation of the KpSC is challenging owing to the close phenotypic and genotypic identity (93–95% average nucleotide identity) shared between these members. Current diagnostic assays either fail to detect and identify all KpSC members or misidentify some KpSC members as *K. pneumoniae sensu stricto*. It is currently estimated that ~20% of human infections are caused by members of the KpSC other than *K. pneumoniae*. This leads to underreporting of some KpSC members in both clinical and environmental settings, which impacts our understanding of the importance of each species. Furthermore, it limits our understanding of the global and local epidemiological impact of some members of the KpSC. In this study, a rapid multiplex real-time PCR assay (KpSC-ID) was designed and developed to detect all KpSC members while simultaneously identifying the predominant human pathogens *K. pneumoniae*, *K. quasipneumoniae* and *K. variicola*. Assay performance was verified *in silico* using a panel of over 1,000 publicly available genome sequences and experimentally validated using a panel of genomic DNA extracted from 54 *Enterobacteriaceae*. The assay displayed excellent specificity against over 1,000 genome sequences tested *in silico*. During *in vitro* validation, the pan-KpSC assay detected each (29/29) KpSC species and strains tested. For the species-specific assays, 100% specificity was demonstrated in the *K. pneumoniae*, *K. quasipneumoniae* and *K. variicola* assays, respectively. Sensitivity of 10 genomic equivalents was demonstrated for each assay. Ultimately, the diagnostic assay developed in this study can improve our understanding of the significance of KpSC members, which is important when investigating their routes of transmission and epidemiology.

## Graphical Abstract

Multiplex real-time PCR to detect the *Klebsiella pneumoniae* species complex and specifically identify *Klebsiella pneumoniae*, *Klebsiella quasipneumoniae* and *Klebsiella variicola*.

**Impact Statement**

In this study, we have developed and optimised the first diagnostic real-time PCR assay, KpSC-ID, which is capable of simultaneously detecting all members of the KpSC, which also specifically detects and identifies the three most common and prevalent human pathogens in the species complex: *Klebsiella pneumoniae*, *Klebsiella variicola* and *Klebsiella quasipneumoniae*. This research addresses a critical gap in our current understanding of the KpSC; despite the clinical importance of the KpSC, the true burden of infection caused by KpSC members other than *K. pneumoniae* remains limited. The KpSC-ID assay developed in this study offers a rapid and scalable method to conduct large-scale retrospective and prospective investigations into KpSC occurrence, spread and prognosis. KpSC-ID has the potential to be used as a tool for improving diagnostic accuracy, informing antimicrobial treatment decisions and enhancing surveillance of healthcare-associated infections. It is also important for understanding the true epidemiology and importance of each member of the KpSC in a range of settings. As such, KpSC-ID is also of interest in non-clinical research settings as a method to identify and screen for members of the KpSC from food, animal and environmental sources.

## Data Availability

The authors confirm that all supporting data, code and protocols have been provided within the article or through supplementary data files.

## Introduction

*Klebsiella* species are ubiquitous in the environment and are the third most frequently reported cause of healthcare-associated infection [1,2]. They cause an estimated 7440 deaths in the European Union/European Economic Area per year and are associated with 25% of the antimicrobial resistance-associated disability-adjusted life years [3]. *Klebsiella pneumoniae* forms part of the ESKAPE pathogens – a group of bacterial species which are considered the most burdensome causes of healthcare-associated infection [4]. Although they are important human pathogens, methods used to routinely discriminate between *Klebsiella* species are imprecise [5]. Several species in the genus share 93–95% average nucleotide identity to * K. pneumoniae sensu stricto*, forming a monophyletic clade known as the *K. pneumoniae* species complex (KpSC) [6]. These species were initially considered to be phylogroups of *K. pneumoniae sensu stricto* herein referred to as *K. pneumoniae* [7]. The recognition of these phylogroups as separate species has thus altered our understanding of the role of *K. pneumoniae* and its associated species complex in human infection [8]. Currently, the KpSC consists of *K. pneumoniae* (previously phylogroup Kp1), *K. quasipneumoniae* subsp. *quasipneumoniae* (Kp2) and subsp. *similipneumoniae* (Kp4), *K. variicola* subsp. *variicola* (Kp3) and subsp. *tropica* (Kp5), *K. quasivariicola* (Kp6) and *K. africana* (Kp7) [6].

The close genetic relatedness of the KpSC members makes accurate identification difficult [5]. Culture-based diagnostics are unable to differentiate between species. Existing molecular diagnostic methods predate many of the taxonomic updates in the KpSC and thus may not be accurate methods of identification. MALDI-TOF MS is unable to identify each KpSC with full specificity, either due to spectral datasets providing inaccurate species information or a lack of unique mass profiles for each member [9,10]. This has resulted in a lack of reliable data concerning their role in infection and overall epidemiology. Since the earliest studies of the KpSC, it has been suggested that *K. pneumoniae*, or Kp1, causes the majority (80%) of healthcare-associated infections [11]. This figure ranges between 65 and 96% in more recent studies [12,14]. *K. variicola* is typically the second most frequently isolated, at 4–25%, followed by *K. quasipneumoniae* at 1.5–8% [9,12, 13, 15,19]. The level of antimicrobial resistance (AMR) amongst members of the KpSC also varies considerably. Of the 944 isolates submitted to the European Study on Carbapenemase-Producing Enterobacteriaceae study as ‘carbapenem-non-susceptible’, 99.5% (*n*=939) were *K. pneumoniae* (*K. variicola=*0.3%, *n=*3; *K. quasipneumoniae*=0.2%, *n*=2) [14]. This suggests that amongst European KpSC lineages, almost all isolates displaying carbapenem resistance are *K. pneumoniae*.

There are other studies which suggest that levels of AMR, disease prognosis and patient outcomes may differ amongst the KpSC. Investigations into species-specific outcomes of KpSC infection have produced varying results. A recent prospective study of bloodstream infection (BSI) caused by KpSC in Norway reported a significantly higher adjusted hazard ratio for monomicrobial BSI caused by *K. variicola* relative to *K. pneumoniae* (*n*=829, *P*=0.02) [20]. Multilocus sequence typing on BSI isolates in Sweden showed that 30-day mortality was higher for patients infected with *K. variicola* in a multivariate analysis adjusted for patient comorbidities (29.4% vs. 13.5%; *P=*0.037) [12]. Imai *et al.* [16] found that *K. variicola* and *K. quasipneumoniae* were associated with older (>80 years) patients and those with malignancy, respectively. In that study, 27% (*n=*119) of BSIs attributed to * K. pneumoniae* were demonstrated to be other KpSC members. For military personnel with trauma-related injuries, it was found that *K. variicola* was associated with bacteraemia and that levels of multidrug resistance (MDR) in *K. pneumoniae* were considerably higher [21]. There were no significant differences between clinical characteristics or disease complications in a study investigating KpSC isolates from urine [13]; however, the authors also found that *K. pneumoniae* had higher levels of resistance to several types of antimicrobials.

Relative rates of each KpSC species/member, geographic location, patient comorbidity, infectious site and local AMR pattern may affect the outcomes of studies such as those mentioned above. In addition, many investigations into the KpSC or *K. pneumoniae* are restricted to bloodstream or MDR isolates [22,24]. This may result in disproportionate representation of certain species-specific outcomes. There is, therefore, a need to investigate KpSC from a diverse range of clinical and environmental sample types [8]. The ability to differentiate members of the KpSC rapidly and accurately would improve our understanding of their epidemiology, transmission and resistance patterns.

Due to the challenges associated with identifying KpSC using culture and/or molecular-based methods, we sought to develop a rapid, culture-free diagnostic assay with improved capability for species identification. A series of novel real-time PCR assays were developed to specifically detect (i) the KpSC, (ii) *K. pneumoniae*, (iii) *K. variicola* and (iv) *K. quasipneumoniae.* These assays were then combined to create a multiplex real-time PCR, herein referred to as KpSC-ID, which can be used to simultaneously detect and differentiate between the most frequently reported members of the KpSC.

## Methods

### Bacterial strains and culture conditions

A panel of 54 *Klebsiella* and other closely related *Enterobacteriaceae* was used to verify the specificity of the multiplex real-time PCR developed in this study. For the KpSC panel tested (*n*=29), the type strain and/or other representative species were obtained from culture collections that had previously been characterised. Recommended growth conditions from the originating culture collection were followed (Table 1). Each strain was grown on nutrient agar (LabM), tryptone soya agar (Oxoid) or tryptone soya agar with the addition of 0.3% (w/v) yeast extract (Oxoid) for 24 h or until sufficient growth was observed. A single colony was then transferred to the equivalent liquid medium and incubated for a further 18 h with continuous shaking at 120 r.p.m.

**Table 1.** Growth conditions and details of culture collection strains used for specificity testing

Species	Subspecies	ID*	KpSC†	Growth medium‡	Temp. (°C)
*K. pneumoniae*	*pneumoniae*	CIP 104216	1	TS	37
*K. pneumoniae*	*pneumoniae*	DSM 681	1	N	37
*K. pneumoniae*	*pneumoniae*	DSM 2026	1	N	37
*K. pneumoniae*	*pneumoniae*	DSM 5758	1	N	37
*K. pneumoniae*	*pneumoniae*	DSM 9377	1	N	37
*K. pneumoniae*	*pneumoniae*	DSM 9376	1	N	37
*K. pneumoniae*	*pneumoniae*	DSM 11678	1	N	37
*K. pneumoniae*	*pneumoniae*	DSM 12059	1	N	37
*K. pneumoniae*	*pneumoniae*	DSM 16609	1	N	37
*K. pneumoniae*	*pneumoniae*	DSM 30102	1	N	37
*K. pneumoniae*	*pneumoniae*	DSM 30104^T^	1	N	37
*K. pneumoniae*	*ozaenae*	DSM 16358	1	N	37
*K. pneumoniae*	*rhinoscleromatis*	DSM 16231	1	N	37
*K. quasipneumoniae*	*quasipneumoniae*	CIP 111839	2	TS	37
*K. quasipneumoniae*	*quasipneumoniae*	CIP 111852	2	TS	37
*K. quasipneumoniae*	*quasipneumoniae*	DSM 28211^T^	2	N	37
*K. variicola*	*variicola*	ATCC 31488	3	N	37
*K. variicola*	*variicola*	CIP 108642	3	TS	30
*K. variicola*	*variicola*	DSM 15968^T^	3	TS	37
*K. quasipneumoniae*	*similipneumoniae*	CIP 111862	4	TS	30
*K. quasipneumoniae*	*similipneumoniae*	CIP 111869	4	TS	30
*K. quasipneumoniae*	*similipneumoniae*	DSM 28212^T^	4	N	37
*K. quasipneumoniae*	*similipneumoniae*	DSM 100662	4	N	37
*K. variicola*	*tropica*	CIP 111654^T^	5	TS	30
*K. variicola*	*tropica*	CIP 111870	5	TS	30
*Klebsiella variicola*	*tropica*	CIP 111898	5	TS	37
*K. quasivariicola*		CIP 111871	6	TS	30
*K. quasivariicola*		CIP 111897	6	TS	37
*K. africana*		CIP 111653^T^	7	TS	30
*Klebsiella aerogenes*		DSM 12058		TS	37
*Klebsiella aerogenes*		DSM 30053		TS	37
*Klebsiella aerogenes*		LMG 2969		TS	37
*Klebsiella grimontii*		CIP 111401		TS	37
*Klebsiella michiganensis*		CIP 110787		TS	30
*Klebsiella ornithinolytica*		DSM 7464		N	30
*Klebsiella oxytoca*		ATCC 43086		N	37
*Klebsiella oxytoca*		DSM 5175		N	37
*Klebsiella pasteurii*		CIP 111696		TS	37
*Klebsiella planticola*		CIP 100751		TS	30
*Klebsiella planticola*		DSM 2688		N	30
*Klebsiella planticola*		DSM 4617		N	30
*Klebsiella terrigena*		DSM 2687		N	30
*Klebsiella terrigena*		DSM 7331		N	30
*Enterobacter cloacae*		ATCC 13047		TS	37
*Escherichia coli*		DSM 30083		TS	37
*Kluyvera intermedia*		CIP 79.27		TS	30
*Leclercia adecarboxylata*		DSM 5077		N	37
*Lelliottia amnigena*		DSM 4486		TS and 0.3% YE	30
*Pantoea agglomerans*		NCTC 9381		TS	30
*Pluralibacter gergoviae*		ATCC3 3028		TS	37
*Serratia marcescens*		DSM 30126		N	30

*Culture collection identification number.

†*K. pneumoniae* species complex phylogroup.

‡Growth medium: TS, tryptone soya; N, nutrient; TS & 0.3% YE, tryptone soya & 0.3% w/v yeast extract.

### Extraction and quantification of genomic DNA

Genomic DNA (gDNA) was extracted from liquid culture using the Zymo Quick gDNA Miniprep Kit (Zymo Research) following the manufacturer’s protocol for ‘Cell Suspensions and Proteinase K Digested Samples’. Liquid culture (1 ml) was pelleted, and the supernatant discarded. Zymo Genomic Lysis buffer (800 µl) was added directly to the pellet. The pellet was resuspended with vortexing and incubated at room temperature for 10 min. The manufacturer’s protocol was followed thereafter until the final elution step. gDNA was eluted using two 50 µl additions of nuclease-free water (Ambion, Thermo Fisher) with a 2-min incubation at room temperature and centrifugation for 30 s at 21,300 ***g*** following each addition, to give a final elution volume of 100 µl. A Qubit 1.0 fluorometer and High Sensitivity dsDNA Kit (Thermo Fisher) were used to quantify eluted gDNA. Samples were stored at −20 °C prior to use.

### Nucleic acid diagnostic target identification

Nucleic acid diagnostic (NAD) targets for the real-time PCR assays developed in this study were identified by evaluating genes that are conserved in *Klebsiella* or have previously been used in NAD assays [25,27]. FASTA sequence alignments were created using the clustal omega tool from the European Bioinformatics Institute [28]. Diagnostic motifs such as insertions and deletions, regions of nucleotide conservation or SNPs were identified. If these features were uniquely present in KpSC, the NAD target was further evaluated.

Nucleotide sequence alignments of NAD targets deemed suitable for KpSC differentiation were investigated for regions in which primers and probes could be developed. The accession numbers of the 348 nt sequences, which were aligned and used for *in silico* assay development, can be found on the web only (Table S1, available in the online Supplementary Material).

Novel real-time PCR assays were created to specifically detect the KpSC (Kp1-Kp7), *K. pneumoniae* (Kp1), *K. quasipneumoniae* (Kp2 and Kp4) and *K. variicola* (Kp3 and Kp5). Oligonucleotide primers and probes were designed manually in accordance with general recommendations and guidelines [29] (Table 2). Particular emphasis was placed on the design of putative probes when evaluating potential diagnostic targets. A minimum of two mismatches within a potential probe region for the target of interest or alternatively an insertion or deletion, which would enable specific probe design, was an essential design criterion. A non-competitive internal amplification control (IAC) [30] assay was designed and included in the multiplex. This assay targets a 500 bp synthetic gene fragment (Table S2). The primers and probe for the IAC were manually designed following the same guidelines as above. All oligonucleotide primers and probes used in this study were manufactured by Integrated DNA Technologies.

**Table 2.** The sequence, target and concentrations of oligonucleotide primers and probes developed for the real-time multiplex PCR assay to identify members of the KpSC

Oligonucleotide	Function	Target	Sequence (5′−3′)*	Final conc. (µM)
Kqp_F	*K. quasipneumoniae* forward primer	rapZ	TGTCGAGCAAGAATTTGTC	0.500
Kqp_P	*K. quasipneumoniae* probe		**HEX**/ATTGTCGAT/**ZEN**/ACCTCGGAGATGTC/Iowa Black FQ	0.200
Kqp_R	*K. quasipneumoniae* reverse primer		GAAGGATTCGAAGACCATG	0.500
Kpneu_F	*K. pneumoniae* forward primer	lepA	GCTGAACTTTATCGACACC	0.500
Kpneu_P	*K. pneumoniae* probe		**CYAN500**/AGATGGATCTCGAAGTGGTTCC/Iowa Black FQ	0.200
Kpneu_R1	*K. pneumoniae* reverse primer		CAGGTCGATCTTGTTGAG	0.250
Kpneu_R2	*K. pneumoniae* reverse primer		GCAGGTCGATCTTATTGAG	0.250
Kvarii_F1	*K. variicola* forward primer	lepA	TCAGCAGTTCGATATCGC	0.500
Kvarii_F2	*K. variicola* forward primer		AAGCTGCTGCAGAAGC	0.500
Kvarii_P	*K. variicola* probe		**FAM**/CGGCAAATA/**ZEN**/ATTAAGGAGTTGG/Iowa BlackFQ	0.200
Kvarii_R	*K. variicola* reverse primer		TCGCAATGACCAAAATCAG	0.500
ZKIR_F	KpSC forward primer	ZKIR	ATCTGTTACTTTATCACGTTCC	1.000
ZKIR_P1	KpSC probe 1		**ROX**/ATGCTATATCTGAAGTGTCTCATTTTCG/Iowa Black RQ	0.400
ZKIR_P2†	KpSC probe 2		**ROX**/TGCTATATCTGAAATGTCTCATTTTCGG/Iowa Black RQ	0.400
ZKIR_R	KpSC reverse primer		AGTGGCGAATGCAATCA	1.000
IAC_F	IAC primer	Synthetic construct	ATGCCAGTCAGCATAAGGA	0.250
IAC_P	IAC probe		**Cy5**/TCGGCACTA/**TAO**/CCGACACGAAC/Iowa Black RQ	0.200
IAC_R	IAC primer		CAGACCTCTGGTAGGATGTAC	0.250

*Flurophores and quenchers are shown in bold; HEX, 4,4,7,2,4,5,7-hexachloro-6-carboxyfluorescein; CYAN500, CYAN500 *N-hydroxysuccinimide* ester; FAM, 6-carboxyfluorescein; ROX, carboxy-X-rhodamine; Cy5, indodicarbocyanine 5. Iowa Black®, ZEN™ and TAO™ are non-abbreviated trademarks.

†Required for the detection of *K. africana* (Kp7).

### Optimisation of multiplex real-time PCR reaction

Real-time PCR experiments were performed using a Roche LightCycler® 480 System II and the LightCycler® 480 Probes Master Kit (Roche Diagnostics). Assay performance was initially investigated in monoplex format. Prior to multiplexing, a colour compensation file was generated per the manufacturer’s instructions and applied to each subsequent multiplex PCR experiment.

Optimum reaction conditions (thermocycling parameters, oligonucleotide concentrations) for multiplex real-time PCR were determined empirically, using 1×10^4^ Genome Equivalents (GE) of gDNA as template. Genome size was calculated based on the genome of *K. pneumoniae*, DSM30104^T^, *K. quasipneumoniae* DSM28211^T^, *K. variicola* DSM 15968^T^ and *K. africana* CIP111653^T^ [6]. The optimised PCR master mix contained 2X LightCycler® 480 Probes Master, primers and probes as outlined in Table 2, template DNA (5 µl) and IAC template (500 copies), adjusted to a final volume of 20 µl with nuclease-free water (Ambion, Thermo Fisher). Following template addition, the sealed plates were incubated at 55 °C with shaking at 180 rpm for 5 min (Thermomixer® C, Eppendorf) prior to thermocycling. The real-time PCR thermocycling parameters used were as follows: initial enzyme activation for 10 mins at 95 °C, 50 cycles of 95 °C for 10 s and 63 °C for 30 s and a final single cooling step of 40 °C for 10 s. The temperature transition rate was 4.4 °C/s while heating and 2.2 °C/s while cooling.

### Determination of specificity and analytical sensitivity

*In vitro* specificity was tested in both monoplex and multiplex formats using 1×10^4^ GE of gDNA from each of the 54 strains outlined in Table 1. A log_10_ dilution series of *K. pneumoniae* DSM30104^T^, *K. quasipneumoniae* DSM28211^T^ and *K. variicola* DSM15968 ^T^ from 1×10^4^ GE to 10 GE was used to determine the analytical sensitivity of each assay in the multiplex.

### Expanded *in silico* evaluation of various sequence types

Owing to the genomic diversity within members of the KpSC, an additional evaluation was performed to determine if the developed diagnostic assays would detect a wide range of KpSC sequence types (STs). Previous studies have identified ~1,000 STs within the KpSC [26,31]. Based on these studies, 1,001 genomes were analysed for each of the 4 assays developed in this study (Table S3). A total of 10 sequences were in addition used for *K. quasivariicola* and *K. africana*, as these members of the KpSC have limited ST diversity recorded at this time. In total, 992 different STs were tested.

To perform this evaluation, the amplicon region for each of the targets (primers, probes and intervening sequence) was blast analysed against the Pasteur ID number (Table S3) on the BIGSdb-Pasteur database (https://bigsdb.pasteur.fr/klebsiella/). This publicly available database is a genome-based strain taxonomy and nomenclature platform of the Institut Pasteur. Once the data were returned, FASTA files were then downloaded and alignments performed using clustal omega.

## Results

### Nucleic acid diagnostic target identification

Regions of the *Klebsiella* genome that have previously been shown to be conserved or to act as suitable NAD targets were evaluated. For the assay to detect all KpSC members, a previously described intergenic region, referred to as the ZKIR (*zur-khe* intergenic region), was evaluated [26]. The forward primer and probe were located within this region, and to enhance specificity, the reverse primer was located downstream at the beginning of the *khe* (annotated as a putative haemolysin) gene. The sequence similarity spanning the ZKIR region to the reverse primer demonstrated 84.04–96.31% identity. Nucleotide conservation in ZKIR meant that it was not possible to design species-specific primers and probes in this region. Alternative NAD targets, namely the genes encoding elongation factor 4 (*lepA*) and the small RNA-binding protein RapZ (*rapZ*) [25,32], were found to contain sufficient nucleotide heterogeneity to differentiate *K. pneumoniae*, *K. quasipneumoniae* and *K. variicola.* For the *K. pneumoniae* assay *lepA* was evaluated and demonstrated 92.61–98.56% nucleotide sequence identity between members of the KpSC. For the *K. quasipneumoniae* assay, the *rapZ* gene was evaluated which demonstrated 91.58–98.6% nucleotide sequence identity between KpSC members. For the *K. quasipneumoniae* assay, the *lepA* gene was also utilized along with its intergenic region and the beginning of *lepB*. The nucleotide sequence identity between KpSC members in this region was 92.54–98.6%, respectively.

### Design of primers and probes for real-time PCR

Primers and probes that targeted conserved regions in the KpSC, *K. pneumoniae*, *K. quasipneumoniae* and *K. variicola* were designed (Table 2). For the KpSC assay, two probes (ZKIR_P2, Table 2) were required for optimal specificity. For the *K. pneumoniae* assay, 2–5 mismatches were present in the probe region to differentiate *K. pneumoniae* from other KpSC members. For the * K. quasipneumoniae* assay, 3-4 mismatches were present in the probe region to differentiate *K. quasipneumoniae* from other KpSC members. Finally, for the *K. variicola* assay, the probe was located in the intergenic region between the *lepA* and *lepB* genes*.* In this region, a 2 bp deletion specific to *K. variicola* was identified, which enabled the design of a highly specific probe for *K. variicola*. Additional primers were also required in the *K. pneumoniae* (Kpneu_R2, Table 2) and *K. variicola* (Kvarii_F2, Table 2) assays to increase analytical sensitivity for a small number of strains. *In silico* analysis of 348 sequences determined that all assays were 100% specific for their intended target micro-organisms.

### Validation of assay specificity and analytical sensitivity

*In vitro* validation using gDNA extracted from a panel of 54 culture collection isolates (Table 1) was performed in monoplex and multiplex formats. Both monoplex and multiplex assays were found to be 100% specific for the target group or species, for the 54 strains tested. They demonstrated 100% concordance with strain designations from culture collections.

The results interpretation matrix for the discrimination of *K. pneumoniae*, *K. variicola* and *K. quasipneumoniae* and the presumptive identification of *K. quasivariicola* and *K. africana* is shown in Table 3. A positive result in the Cy5 IAC channel must be observed for a result to be considered valid. A positive result in the ROX channel indicates that a KpSC member is present in the sample. An additional positive in the CYAN500, FAM or HEX channels identifies the species as *K. pneumoniae*, *K. variicola* or *K. quasipneumoniae*, respectively (Fig. S1).

**Table 3.** Multiplex results interpretation for the detection of KpSC

CYAN 500	FAM	HEX	ROX	Cy5	Result: species (phylogroup)
440– 488 nm*	498–580 nm*	533–580 nm*	533–610 nm*	618–660 nm*,†	
+	–	–	–	+/−	*K. pneumoniae* (Kp1)‡
–	+	–	+	+/−	*K. variicola* (Kp3/Kp5)‡
–	–	+	+	+/−	*K. quasipneumoniae* (Kp2/Kp4)‡
–	–	–	+	+/−	Kp6 or Kp7§
–	–	–	–	+	Negative for KpSC
–	–	–	–	–	Invalid IAC (repeat)¶

*Excitation and emission filters used on LightCycler® 480.

†In the presence of high concentrations of primary target, the IAC may become inhibited.

‡Detection in the species-specific identifying channel (Cyan 500, FAM or HEX) and ROX is required for the result to be considered valid.

§Detection of KpSC but not Kp1-Kp5 indicates the presence of *K. quasivariicola* (Kp6) or *K. afriana* (Kp7). Requires confirmatory testing.

¶The result is considered invalid and should be repeated.

The analytical sensitivity was assessed using a log_10_ dilution of quantified gDNA from the type strain of each target species. Using the optimised reaction conditions described, analytical sensitivity of 10 GE was achieved for each target strain tested (Fig. S2).

### Expanded *in silico* evaluation of various sequence types

Genomes (*n*=1,001) were analysed for the four assays developed in this study. These genomes represented 992 different STs of KpSC (*K. pneumoniae*, *n*=705; *K. quasipneumoniae* subsp. *quasipneumoniae*, *n*=49; *K. variicola* subsp. *variicola*, *n*=149; * K. quasipneumoniae* subsp. *similipneumoniae*, *n*=64; *K. variicola* subsp. *tropica*, *n*=13; *K. quasivariicola*, *n*=8; and *K. africana*, *n*=4). Additional sequences for *K. quasivariicola* and *K. africana* were chosen at random to ensure a minimum of 10 sequences were included for all KPSC (Table S3).

The region of the gene amplified and detected for each of the four assays was blast searched against these 1001 genomes. For the KpSC assay, over 99% (992/1001) of nucleotide sequences analysed demonstrated 100% sequence identity in all primer and probe regions. For 9/1001 sequences analysed, a mismatch was present in the reverse primer site (Table S3).

For the *K. pneumoniae* assay, 705 STs were analysed for inclusivity testing. From this analysis, ~90% (639/705) of the *K. pneumoniae* STs demonstrated 100% sequence identity with all primers and probes. In 61 s STs, 1 mismatch was present in the reverse primer. In 3 STs (ST3661, ST3607, ST610), 1 mismatch was present in the reverse primer site and 1 mismatch was present in the probe region, respectively. In 2 STs (ST3708, ST2958), a mismatch was present in the probe alone. One *K. pneumoniae* ST (ST715) demonstrated significant sequence heterogeneity in the probe region and would likely not be detected by this assay (false negative). Of the 296 other KpSC STs and sequences analysed here, 1 *K. variicola* subsp. *variicola* ST (ST3952) would likely result in a false positive, as it demonstrated 100% sequence similarity in the assay region (Table S3). For the remaining 295 STs, a minimum of 2 mismatches were present in the probe region, and as such, no other false positive results are predicted.

For the *K. variicola* assay, 162 STs were analysed for inclusivity. From this analysis, 161 STs demonstrated 100% sequence identity to the primer and probe regions. One *K. variicola* subsp. *tropica* ST (ST2600) had 1 mismatch in the reverse primer. The probe region, which contains a 2 bp deletion unique to *K. variicola* subsp. *variicola* and subsp. *tropica*, was not deleted in the other 839/1,001 STs and sequences analysed and as such no false positives are predicted.

For the *K. quasipneumoniae* assay, 113 STs were analysed for inclusivity. From this analysis, ~95% of *K. quasipneumoniae* STs demonstrated 100% identity to all primer and probe regions. In 5/49 *K. quasipneumoniae* subsp. *quasipneumoniae* STs, a single mismatch was present in the probe region. In *K. quasipneumoniae* subsp. *similipneumoniae*, 1 ST had 1 mismatch in the forward primer. For the 888/1,001 STs and sequences analysed, 3–4 mismatches were present in the probe region alone, and as such, no false positive results are predicted.

## Discussion

Currently, accurate KpSC differentiation relies on whole-genome sequence analysis [6,17]. We have developed an internally controlled multiplex real-time PCR assay which enables pan-KpSC detection and the simultaneous identification of the three most clinically relevant species: *K. pneumoniae*, *K. quasipneumoniae* and *K. variicola.* The assay specificity has been tested *in silico* using over 1,000 unique nucleotide sequences (Tables S1 and S3) and *in vitro* using a panel of 54 *Klebsiella* and *Enterobacteriaceae* (Table 1). This is the first multiplex real-time PCR assay with the ability to distinguish the three main members of the KpSC. The present novel assay has been validated *in vitro* using all 7 KpSC phylogroups. In addition to the identification of *K. pneumoniae*, *K. quasipneumoniae* and *K. variicola,* this assay can also be used for the presumptive identification of *K. quasivariicola* and * K. africana* by combining positivity for KpSC and negativity for the three main species (Table 3).

NAD assays for the detection of KpSC members have previously been described, but many of these studies are limited by the use of incomplete strain panels for specificity testing. A real-time PCR assay targeting the *fiu* gene to detect but not differentiate the species *K. pneumoniae*, *K. quasipneumoniae* and *K. variicola* was described by Sun *et al.* [33]. However, no other KpSC members were included in the exclusivity panel. A series of monoplex hydrolysis probe-based real-time PCR assays were described by Dereeper *et al.* [34] to detect Kp1–Kp6. These assays have not been tested using *K. africana* (Kp7) and lack the advantage of being multiplexed into a single assay. The simultaneous detection of all KpSC members using real-time PCR is possible through the use of a SYBR® Green-based assay targeting the intergenic region referred to as ‘ZKIR’ [26]. Although the assay provides a useful means of detecting the KpSC, it does not differentiate between species. A recently described triplex real-time PCR assay, which targets the ZKIR to detect the KpSC, has an additional probe to simultaneously identify *K. pneumoniae* [35] and, incidentally, can also detect Kp6. Our study complements this recent publication and further differentiates the KpSC with the ability to specifically identify *K. quasipneumoniae* or *K. variicola*, which are the two main clinical species besides *K. pneumoniae*.

Rapid diagnostics are an essential tool in reducing the spread of healthcare-associated infections and AMR [36]. Improved methods of KpSC identification would increase our understanding of their relative roles in human infection and the environment and their contribution to AMR. As such, we developed a real-time multiplex PCR to detect each member of the KpSC and differentiate between *K. pneumoniae*, *K. quasipneumoniae* and *K. variicola.* The specific detection of these three species will enable the rapid and accurate identification of the three most prominent members of the KpSC. This is combined with the pan-KpSC assay, which can also detect *K. quasivariicola* (Kp6) and *K. africana* (Kp7).

The prevalence of KpSC in the environment and the role they play in clinical settings highlight the need for improved understanding of the epidemiology and transmission of members of the KpSC [37]. KpSC-ID was developed to add to existing literature and methodologies to address this need. KpSC-ID has been validated against 348 sequences *in silico* and has demonstrated 100% specificity when tested against 54 *Enterobacteriaceae.* Furthermore, 100% concordance was demonstrated to culture collection isolates when specifically identifying 29 KpSC isolates. Owing to the high level of genomic diversity present in *Klebsiella* species, an extended *in silico* analysis was performed to demonstrate the robustness of KpSC-ID across a wide range of STs. The results demonstrate that all assays developed, herein, are highly specific for their respective target and are expected to detect most target isolates: the KpSC assay oligonucleotides are 100% conserved in 992/1,001 genomes analysed, and for the *K. pneumoniae* assay, the primers and probes are 100% conserved in over 90% of ST analysed. For a small number of STs, a mismatch was present, but only in a single oligonucleotide primer. This may affect the sensitivity of the assay slightly, and future iterations of this assay may incorporate an additional primer to cover this variant if deemed necessary. One false negative (ST715) and one false positive (ST3952) result are anticipated for the 1 *K. pneumoniae* assay based on this analysis; however, this represents an extremely low rate of incorrect calls (2/1,001). For the *K. variicola* assay ~99% of ST demonstrated 100% conservation in primers and probes with no cross reactions predicted. Finally, for the *K. quasipneumoniae* assay, 95% of ST demonstrated 100% conservation in primers and probe with a single mismatch present in a minority of ST. No cross reactions or false positive results predicted.

This KpSC-ID assay’s diagnostic performance has been thoroughly investigated both *in silico* and *in vitro*. This has demonstrated the potential of this assay to further understand the role each KpSC member plays both in the environment and clinical settings. Further investigation in clinical and environmental laboratories should now be performed to further validate KpSC-ID. We believe its implementation at a large scale will contribute to further understanding the ecology epidemiology and clinical significance of the individual KpSC taxa.

## Supplementary material

10.1099/mic.0.001587 Uncited Supplementary Material 1.

10.1099/mic.0.001587 Uncited Supplementary Material 2.

## Abbreviations

AMR: antimicrobial resistance
BSI: bloodstream infection
gDNA: genomic DNA
GE: genome equivalents
IAC: internal amplification control
KpSC: *Klebsiella pneumoniae* species complex
MDR: multidrug resistance
NAD: nucleic acid diagnostic
STs: sequence types
