# Supplementary material for: KpSC-ID: a multiplex real-time PCR assay for the simultaneous detection of the Klebsiella pneumoniae species complex and specific identification of Klebsiella pneumoniae, Klebsiella quasipneumoniae and Klebsiella variicola
Source: Microbiology (Reading). 2025 Jul 30;171(7):001587. doi: 10.1099/mic.0.001587 (PMC12310337; doi:10.1099/mic.0.001587)
Supplement: Uncited Supplementary Material 1. [file mic-171-01587-s001.pdf]

Supplementary Table S1: NCBI accession numbers of the sequences used to design and validate each novel real-time PCR assay *in silico*

Sequences used to perform *in silico* analysis for assays targeting *lepA*		
**NCBI Accession Number**	**Species**	**URL**
CP020448.2	*Citrobacter braakii*	https://www.ncbi.nlm.nih.gov/nuccore/CP020448.2
CP024819.1	*Citrobacter freundii*	https://www.ncbi.nlm.nih.gov/nuccore/CP024819.1
CP018810.1	*Citrobacter freundii*	https://www.ncbi.nlm.nih.gov/nuccore/CP018810.1
CP012266.1	*Cronobacter dublinensis*	https://www.ncbi.nlm.nih.gov/nuccore/CP012266.1
CP003312.1	*Cronobacter sakazakii*	https://www.ncbi.nlm.nih.gov/nuccore/CP003312.1
CP012257.1	*Cronobacter universalis*	https://www.ncbi.nlm.nih.gov/nuccore/CP012257.1
CP046618.1	*Enterobacter asburiae*	https://www.ncbi.nlm.nih.gov/nuccore/CP046618.1
CP056474.1	*Enterobacter cloacae*	https://www.ncbi.nlm.nih.gov/nuccore/CP056474.1
CP058253.1	*Enterobacter roggenkampii*	https://www.ncbi.nlm.nih.gov/nuccore/CP058253.1
AP019007.1	*Enterobacter oligotrophicus*	https://www.ncbi.nlm.nih.gov/nuccore/AP019007.1
LR134122.1	*Klebsiella aerogenes*	https://www.ncbi.nlm.nih.gov/nuccore/LR134122.1
CP002824.1	*K. aerogenes*	https://www.ncbi.nlm.nih.gov/nuccore/CP002824.1
NC015663.1	*K. aerogenes*	https://www.ncbi.nlm.nih.gov/nuccore/NC015663.1
CP024880.1	*K. aerogenes*	https://www.ncbi.nlm.nih.gov/nuccore/CP024880.1
CP028951.1	*K. aerogenes*	https://www.ncbi.nlm.nih.gov/nuccore/CP028951.1
CP023963.1	*K. aerogenes*	https://www.ncbi.nlm.nih.gov/nuccore/CP023963.1
CP011574.1	*K. aerogenes*	https://www.ncbi.nlm.nih.gov/nuccore/CP011574.1
CP026722.1	*K. aerogenes*	https://www.ncbi.nlm.nih.gov/nuccore/CP026722.1
CP033817.1	*K. aerogenes*	https://www.ncbi.nlm.nih.gov/nuccore/CP033817.1
CP026756.1	*K. aerogenes*	https://www.ncbi.nlm.nih.gov/nuccore/CP026756.1
CP031756.1	*K. aerogenes*	https://www.ncbi.nlm.nih.gov/nuccore/CP031756.1
CP014029.2	*K. aerogenes*	https://www.ncbi.nlm.nih.gov/nuccore/CP014029.2
CP014748.2	*K. aerogenes*	https://www.ncbi.nlm.nih.gov/nuccore/CP014748.2
CP024885.1	*K. aerogenes*	https://www.ncbi.nlm.nih.gov/nuccore/CP024885.1
CP011539.1	*K. aerogenes*	https://www.ncbi.nlm.nih.gov/nuccore/CP011539.1
FO203355.1	*K. aerogenes*	https://www.ncbi.nlm.nih.gov/nuccore/FO203355.1
LR134475.1	*K. aerogenes*	https://www.ncbi.nlm.nih.gov/nuccore/LR134475.1
CP024883.1	*K. aerogenes*	https://www.ncbi.nlm.nih.gov/nuccore/CP024883.1
NZ_CP059391.1	*Klebsiella africana*	https://www.ncbi.nlm.nih.gov/nuccore/NZ_CP059391.1
NZ_CP084874.1	*K. africana*	https://www.ncbi.nlm.nih.gov/nuccore/NZ_CP084874.1
CAAHGQ010000005.1	*K. africana* Kp7 CIP111653	https://www.ncbi.nlm.nih.gov/nuccore/CAAHGQ010000005.1
NZCP044527.1	*Klebsiella grimontii*	https://www.ncbi.nlm.nih.gov/nuccore/NZCP044527.1
CP067433.1	*K. grimontii*	https://www.ncbi.nlm.nih.gov/nuccore/CP067433.1
NZ_LR607336.1	*K. grimontii*	https://www.ncbi.nlm.nih.gov/nuccore/NZ_LR607336.1

CP023185.1	*Klebsiella michiganensis*	https://www.ncbi.nlm.nih.gov/nuccore/CP023185.1
CP004887.1	*K. michiganensis*	https://www.ncbi.nlm.nih.gov/nuccore/CP004887.1
CP003218.1	*K. michiganensis*	https://www.ncbi.nlm.nih.gov/nuccore/CP003218.1
NZAP022547.1	*K. michiganensis*	https://www.ncbi.nlm.nih.gov/nuccore/NZAP022547.1
CP073236.1	*K. michiganensis*	https://www.ncbi.nlm.nih.gov/nuccore/CP073236.1
CP027426.1	*Klebsiella oxytoca*	https://www.ncbi.nlm.nih.gov/nuccore/CP027426.1
CP020358.1	*K. oxytoca*	https://www.ncbi.nlm.nih.gov/nuccore/CP020358.1
CP026285.1	*K. oxytoca*	https://www.ncbi.nlm.nih.gov/nuccore/CP026285.1
CP011636.1	*K. oxytoca*	https://www.ncbi.nlm.nih.gov/nuccore/CP011636.1
JAIFQY010000013.1	*Klebsiella pasteurii*	https://www.ncbi.nlm.nih.gov/nuccore/JAIFQY010000013.1
CP089403.1	*K. pasteurii*	https://www.ncbi.nlm.nih.gov/nuccore/CP089403.1
CABGGT010000018.1	*K. pasteurii* CIP111696	https://www.ncbi.nlm.nih.gov/nuccore/CABGGT010000018.1
CP026160.1	*Klebsiella pneumoniae*	https://www.ncbi.nlm.nih.gov/nuccore/CP026160.1
CP026178.1	*K. pneumoniae*	https://www.ncbi.nlm.nih.gov/nuccore/CP026178.1
CP038002.1	*K. pneumoniae*	https://www.ncbi.nlm.nih.gov/nuccore/CP038002.1
CP036300.1	*K. pneumoniae*	https://www.ncbi.nlm.nih.gov/nuccore/CP036300.1
CP016159.1	*K. pneumoniae*	https://www.ncbi.nlm.nih.gov/nuccore/CP016159.1
LR133964.1	*K. pneumoniae*	https://www.ncbi.nlm.nih.gov/nuccore/LR133964.1
CP021955.1	*K. pneumoniae*	https://www.ncbi.nlm.nih.gov/nuccore/CP021955.1
CP025211.1	*K. pneumoniae*	https://www.ncbi.nlm.nih.gov/nuccore/CP025211.1
CP028816.1	*K. pneumoniae*	https://www.ncbi.nlm.nih.gov/nuccore/CP028816.1
CP015130.1	*K. pneumoniae*	https://www.ncbi.nlm.nih.gov/nuccore/CP015130.1
CP031817.1	*K. pneumoniae*	https://www.ncbi.nlm.nih.gov/nuccore/CP031817.1
CP021696.1	*K. pneumoniae*	https://www.ncbi.nlm.nih.gov/nuccore/CP021696.1
CP014008.1	*K. pneumoniae*	https://www.ncbi.nlm.nih.gov/nuccore/CP014008.1
CP036190.1	*K. pneumoniae*	https://www.ncbi.nlm.nih.gov/nuccore/CP036190.1
CP034082.1	*K. pneumoniae*	https://www.ncbi.nlm.nih.gov/nuccore/CP034082.1
CP035383.1	*K. pneumoniae*	https://www.ncbi.nlm.nih.gov/nuccore/CP035383.1
CP030269.1	*K. pneumoniae*	https://www.ncbi.nlm.nih.gov/nuccore/CP030269.1
CP027189.1	*K. pneumoniae*	https://www.ncbi.nlm.nih.gov/nuccore/CP027189.1
CP025080.1	*K. pneumoniae*	https://www.ncbi.nlm.nih.gov/nuccore/CP025080.1
CP025639.1	*K. pneumoniae*	https://www.ncbi.nlm.nih.gov/nuccore/CP025639.1
CP025631.1	*K. pneumoniae*	https://www.ncbi.nlm.nih.gov/nuccore/CP025631.1
CP025090.1	*K. pneumoniae*	https://www.ncbi.nlm.nih.gov/nuccore/CP025090.1
CP019047.1	*K. pneumoniae*	https://www.ncbi.nlm.nih.gov/nuccore/CP019047.1
CP016813.1	*K. pneumoniae*	https://www.ncbi.nlm.nih.gov/nuccore/CP016813.1
CP014010.1	*K. pneumoniae*	https://www.ncbi.nlm.nih.gov/nuccore/CP014010.1
CP003785.1	*K. pneumoniae*	https://www.ncbi.nlm.nih.gov/nuccore/CP003785.1
AP006725.1	*K. pneumoniae*	https://www.ncbi.nlm.nih.gov/nuccore/AP006725.1
CP026021.1	*K. pneumoniae*	https://www.ncbi.nlm.nih.gov/nuccore/CP026021.1
N/A	*K. pneumoniae* ATCC4352	https://www.atcc.org/products/4352
NZUGLV01000001.1	*K. pneumoniae* ssp. *rhinoscleromatis*	https://www.ncbi.nlm.nih.gov/nuccore/NZUGLV01000001.1

ACZD01000034.1	*K. pneumoniae* ssp. *rhinoscleromatis*	https://www.ncbi.nlm.nih.gov/nuccore/ACZD01000034.1
CP028806.1	*K. pneumoniae*	https://www.ncbi.nlm.nih.gov/nuccore/CP028806.1
CP023478.1	*Klebsiella quasipneumoniae*	https://www.ncbi.nlm.nih.gov/nuccore/CP023478.1
CP014156.1	*K. quasipneumoniae*	https://www.ncbi.nlm.nih.gov/nuccore/CP014156.1
CP014155.1	*K. quasipneumoniae*	https://www.ncbi.nlm.nih.gov/nuccore/CP014155.1
CP014154.1	*K. quasipneumoniae*	https://www.ncbi.nlm.nih.gov/nuccore/CP014154.1
CP012300.1	*K. quasipneumoniae*	https://www.ncbi.nlm.nih.gov/nuccore/CP012300.1
CP012252.1	*K. quasipneumoniae*	https://www.ncbi.nlm.nih.gov/nuccore/CP012252.1
CP031257.1	*K. quasipneumoniae*	https://www.ncbi.nlm.nih.gov/nuccore/CP031257.1
CP029597.1	*K. quasipneumoniae*	https://www.ncbi.nlm.nih.gov/nuccore/CP029597.1
CP014071.1	*K. quasipneumoniae*	https://www.ncbi.nlm.nih.gov/nuccore/CP014071.1
CP014696.2	*K. quasipneumoniae*	https://www.ncbi.nlm.nih.gov/nuccore/CP014696.2
CP034678.1	*K. quasipneumoniae*	https://www.ncbi.nlm.nih.gov/nuccore/CP034678.1
AP019687.1	*K. quasipneumoniae*	https://www.ncbi.nlm.nih.gov/nuccore/AP019687.1
CP039791.1	*K. quasipneumoniae*	https://www.ncbi.nlm.nih.gov/nuccore/CP039791.1
AP022142.1	*K. quasipneumoniae*	https://www.ncbi.nlm.nih.gov/nuccore/AP022142.1
CP084803.1	*K. quasipneumoniae*	https://www.ncbi.nlm.nih.gov/nuccore/CP084803.1
CP030171.1	*K. quasipneumoniae*	https://www.ncbi.nlm.nih.gov/nuccore/CP030171.1
CP029437.1	*K. quasipneumoniae*	https://www.ncbi.nlm.nih.gov/nuccore/CP029437.1
CP029443.1	*K. quasipneumoniae*	https://www.ncbi.nlm.nih.gov/nuccore/CP029443.1
CP029432.1	*K. quasipneumoniae*	https://www.ncbi.nlm.nih.gov/nuccore/CP029432.1
CAAHGF010000008.1	*K. quasipneumoniae* Kp2 CIP111839	https://www.ncbi.nlm.nih.gov/nuccore/CAAHGF010000008.1
CAAHFU010000124.1	*K. quasipneumoniae* Kp2 CIP111852	https://www.ncbi.nlm.nih.gov/nuccore/CAAHFU010000124.1
CAAHFY010000006.1	*K. quasipneumoniae* Kp4 CIP111862	https://www.ncbi.nlm.nih.gov/nuccore/CAAHFY010000006.1
CP084770.1	*K. quasipneumoniae* Kp4 CIP111869	https://www.ncbi.nlm.nih.gov/nuccore/CP084770.1
UJYW01000004.1	*Klebsiella quasivariicola*	https://www.ncbi.nlm.nih.gov/nuccore/UJYW01000004.1
VLPG01000016.1	*K. quasivariicola*	https://www.ncbi.nlm.nih.gov/nuccore/VLPG01000016.1
WHZK01000014.1	*K. quasivariicola*	https://www.ncbi.nlm.nih.gov/nuccore/WHZK01000014.1
UJYZ02000002.1	*K. quasivariicola*	https://www.ncbi.nlm.nih.gov/nuccore/UJYZ02000002.1
UJZG01000020.1	*K. quasivariicola*	https://www.ncbi.nlm.nih.gov/nuccore/UJZG01000020.1
CAAHGS010000079.1	*K. quasivariicola* Kp6 CIP111871	https://www.ncbi.nlm.nih.gov/nuccore/CAAHGS010000079.1
NZ_CAAHGB010000003.1	*K. quasivariicola* Kp6 CIP111879	https://www.ncbi.nlm.nih.gov/nuccore/NZ_CAAHGB010000003.1
CP022823.1	*K. quasivariicola* KPN1705	https://www.ncbi.nlm.nih.gov/nuccore/CP022823.1
NZ_CABEJC010000004.1	*Klebsiella spallanzanii* CIP111695	https://www.ncbi.nlm.nih.gov/nuccore/NZ_CABEJC010000004.1

LR130538.1	*Klebsiella variicola*	https://www.ncbi.nlm.nih.gov/nuccore/LR130538.1
LR134235.1	*K. variicola*	https://www.ncbi.nlm.nih.gov/nuccore/LR134235.1
CP008700.1	*K. variicola*	https://www.ncbi.nlm.nih.gov/nuccore/CP008700.1
CP018307.1	*K. variicola*	https://www.ncbi.nlm.nih.gov/nuccore/CP018307.1
CP000964.1	*K. variicola*	https://www.ncbi.nlm.nih.gov/nuccore/CP000964.1
CP030173.1	*K. variicola*	https://www.ncbi.nlm.nih.gov/nuccore/CP030173.1
CP010523.2	*K. variicola*	https://www.ncbi.nlm.nih.gov/nuccore/CP010523.2
CP001891.1	*K. variicola*	https://www.ncbi.nlm.nih.gov/nuccore/CP001891.1
CP013985.1	*K. variicola*	https://www.ncbi.nlm.nih.gov/nuccore/CP013985.1
LR130543.1	*K. variicola*	https://www.ncbi.nlm.nih.gov/nuccore/LR130543.1
LR130544.1	*K. variicola*	https://www.ncbi.nlm.nih.gov/nuccore/LR130544.1
LR130539.1	*K. variicola*	https://www.ncbi.nlm.nih.gov/nuccore/LR130539.1
CP016344.1	*K. variicola*	https://www.ncbi.nlm.nih.gov/nuccore/CP016344.1
CP028555.1	*K. variicola*	https://www.ncbi.nlm.nih.gov/nuccore/CP028555.1
CP009274.2	*K. variicola*	https://www.ncbi.nlm.nih.gov/nuccore/CP009274.2
CP020847.1	*K. variicola*	https://www.ncbi.nlm.nih.gov/nuccore/CP020847.1
CP017289.1	*K. variicola*	https://www.ncbi.nlm.nih.gov/nuccore/CP017289.1
CP017849.1	*K. variicola*	https://www.ncbi.nlm.nih.gov/nuccore/CP017849.1
CP017284.1	*K. variicola*	https://www.ncbi.nlm.nih.gov/nuccore/CP017284.1
CP032354.1	*K. variicola*	https://www.ncbi.nlm.nih.gov/nuccore/CP032354.1
CP027064.2	*K. variicola*	https://www.ncbi.nlm.nih.gov/nuccore/CP027064.2
CP084767.1	*K. variicola* Kp5	https://www.ncbi.nlm.nih.gov/nuccore/CP084767.1
CAAHGN010000<br>007.1	*K. variicola* Kp5<br>CIP111654	https://www.ncbi.nlm.nih.gov/nuccore/CAAHGN01<br>0000007.1
CAAHGO010000<br>006.1	*K. variicola* Kp5<br>CIP111898	https://www.ncbi.nlm.nih.gov/nuccore/CAAHGO01<br>0000006.1
CAAHGL010000<br>007.1	*K. variicola* Kp5<br>CIP111870	https://www.ncbi.nlm.nih.gov/nuccore/CAAHGL01<br>0000007.1
CP033631.1	*Klebsiella* sp. P1CD1	https://www.ncbi.nlm.nih.gov/nuccore/CP033631.1
CP037441.1	*Klebsiella* sp. PO552	https://www.ncbi.nlm.nih.gov/nuccore/CP037441.1
AP022665.1	*Kluyvera ascorbata*	https://www.ncbi.nlm.nih.gov/nuccore/AP022665.1
CP022114.1	*Kluyvera* genomosp.3	https://www.ncbi.nlm.nih.gov/nuccore/CP022114.1
CP045843.1	*Kluyvera intermedia*	https://www.ncbi.nlm.nih.gov/nuccore/CP045843.1
LR699009.1	*Pluralibacter*<br>*gergoviae*	https://www.ncbi.nlm.nih.gov/nuccore/LR699009.1
CP009450.1	*Pluralibacter*<br>*gergoviae*	https://www.ncbi.nlm.nih.gov/nuccore/CP009450.1
CP002272.1	*Pluralibacter*<br>*lignolyticus*	https://www.ncbi.nlm.nih.gov/nuccore/CP002272.1
CP079752.1	*Raoultella*<br>*ornithinolytica*	https://www.ncbi.nlm.nih.gov/nuccore/CP079752.1
CP008886.1	*R. ornithinolytica*	https://www.ncbi.nlm.nih.gov/nuccore/CP008886.1
CP038281.1	*R. ornithinolytica*	https://www.ncbi.nlm.nih.gov/nuccore/CP038281.1
CP004142.1	*R. ornithinolytica*	https://www.ncbi.nlm.nih.gov/nuccore/CP004142.1
CP012555.1	*R. ornithinolytica*	https://www.ncbi.nlm.nih.gov/nuccore/CP012555.1
LR134195.1	*R. ornithinolytica*	https://www.ncbi.nlm.nih.gov/nuccore/LR134195.1
CP054471.1	*R. ornithinolytica*	https://www.ncbi.nlm.nih.gov/nuccore/CP054471.1
CP029752.1	*Raoultella planticola*	https://www.ncbi.nlm.nih.gov/nuccore/CP029752.1
CP044121.1	*R. planticola*	https://www.ncbi.nlm.nih.gov/nuccore/CP044121.1

CP040183.1	*R. planticola*	https://www.ncbi.nlm.nih.gov/nuccore/CP040183.1
CP019899.1	*R. planticola*	https://www.ncbi.nlm.nih.gov/nuccore/CP019899.1
CP082168.1	*R. planticola*	https://www.ncbi.nlm.nih.gov/nuccore/CP082168.1
CP069815.1	*R. planticola*	https://www.ncbi.nlm.nih.gov/nuccore/CP069815.1
CABDVR010000001.1	*R. planticola*	https://www.ncbi.nlm.nih.gov/nuccore/CABDVR010000001.1
SUPN01000010.1	*R. planticola*	https://www.ncbi.nlm.nih.gov/nuccore/SUPN01000010.1
DADZWO010000013.1	*R. planticola*	https://www.ncbi.nlm.nih.gov/nuccore/DADZWO010000013.1
PDLC01000002.1	*R. planticola*	https://www.ncbi.nlm.nih.gov/nuccore/PDLC01000002.1
CP023877.1	*R. planticola*	https://www.ncbi.nlm.nih.gov/nuccore/CP023877.1
CP023874.1	*R. planticola*	https://www.ncbi.nlm.nih.gov/nuccore/CP023874.1
MPYJ04000036.1	*R. planticola*	https://www.ncbi.nlm.nih.gov/nuccore/MPYJ04000036.1
NZ_JMPP01000022.1	*R. planticola* CIP100751	https://www.ncbi.nlm.nih.gov/nuccore/NZ_JMPP01000022.1
CP016032.1	*Serratia marcescens*	https://www.ncbi.nlm.nih.gov/nuccore/CP016032.1
MH460883.1	*Serratia marcescens*	https://www.ncbi.nlm.nih.gov/nuccore/MH460883.1
CP038662.1	*Serratia nematodiphila*	https://www.ncbi.nlm.nih.gov/nuccore/CP038662.1

Sequences used to perform *in silico* analysis for assay targeting ZKIR

NCBI Accession Number	Species	URL
CP020448.2	*Citrobacter braakii*	https://www.ncbi.nlm.nih.gov/nuccore/CP020448.2
CP024819.1	*Citrobacter freundii*	https://www.ncbi.nlm.nih.gov/nuccore/CP024819.1
CP012253.1	*Cronobacter sakazakii*	https://www.ncbi.nlm.nih.gov/nuccore/CP012253.1
CP012257.1	*Cronobacter universalis*	https://www.ncbi.nlm.nih.gov/nuccore/CP012257.1
CP045769.1	*Enterobacter cancerogenus*	https://www.ncbi.nlm.nih.gov/nuccore/CP045769.1
CP025225.1	*Enterobacter cancerogenus*	https://www.ncbi.nlm.nih.gov/nuccore/CP025225.1
CP056776.1	*Enterobacter cloacae*	https://www.ncbi.nlm.nih.gov/nuccore/CP056776.1
CP041925.1	*K. aerogenes*	https://www.ncbi.nlm.nih.gov/nuccore/CP041925.1
CP055904.1	*K. aerogenes*	https://www.ncbi.nlm.nih.gov/nuccore/CP055904.1
LR134475.1	*K. aerogenes*	https://www.ncbi.nlm.nih.gov/nuccore/LR134475.1
CP035466.1	*K. aerogenes*	https://www.ncbi.nlm.nih.gov/nuccore/CP035466.1
LR134126.1	*K. aerogenes*	https://www.ncbi.nlm.nih.gov/nuccore/LR134126.1
CP047669.1	*K. aerogenes*	https://www.ncbi.nlm.nih.gov/nuccore/CP047669.1
CP045870.1	*K. aerogenes*	https://www.ncbi.nlm.nih.gov/nuccore/CP045870.1
CP044214.1	*K. aerogenes*	https://www.ncbi.nlm.nih.gov/nuccore/CP044214.1
AP022108.1	*K. aerogenes*	https://www.ncbi.nlm.nih.gov/nuccore/AP022108.1
WMHU01000001.1	*K. aerogenes*	https://www.ncbi.nlm.nih.gov/nuccore/WMHU01000001.1
NEYW02000018.1	*K. aerogenes*	https://www.ncbi.nlm.nih.gov/nuccore/NEYW02000018.1

PXKX01000023.1	*K. aerogenes*	https://www.ncbi.nlm.nih.gov/nuccore/PXKX01000023.1
JAKCJA010000021.1	*K. aerogenes*	https://www.ncbi.nlm.nih.gov/nuccore/JAKCJA010000021.1
CP059391.1	*K. africana* Kp7	https://www.ncbi.nlm.nih.gov/nuccore/CP059391.1
CP084874.1	*K. africana* Kp7	https://www.ncbi.nlm.nih.gov/nuccore/CP084874.1
CAAHGQ010000004.1	*K. africana* Kp7 CIP111653	https://www.ncbi.nlm.nih.gov/nuccore/CAAHGQ010000004.1
CP044527.1	*K. grimontii*	https://www.ncbi.nlm.nih.gov/nuccore/CP044527.1
LR607363.1	*K. grimontii*	https://www.ncbi.nlm.nih.gov/nuccore/LR607363.1
LR594038.1	*K. grimontii*	https://www.ncbi.nlm.nih.gov/nuccore/LR594038.1
CP056150.1	*K. grimontii*	https://www.ncbi.nlm.nih.gov/nuccore/CP056150.1
CP079754.1	*K. grimontii*	https://www.ncbi.nlm.nih.gov/nuccore/CP079754.1
CP067433.1	*K. grimontii*	https://www.ncbi.nlm.nih.gov/nuccore/CP067433.1
CP036175.1	*Klebsiella huaxiensis*	https://www.ncbi.nlm.nih.gov/nuccore/CP036175.1
CP003218.1	*K. michiganensis*	https://www.ncbi.nlm.nih.gov/nuccore/CP003218.1
CP048108.1	*K. michiganensis*	https://www.ncbi.nlm.nih.gov/nuccore/CP048108.1
LR607380.1	*K. michiganensis*	https://www.ncbi.nlm.nih.gov/nuccore/LR607380.1
CP071393.1	*K. michiganensis*	https://www.ncbi.nlm.nih.gov/nuccore/CP071393.1
LR607366.1	*K. michiganensis*	https://www.ncbi.nlm.nih.gov/nuccore/LR607366.1
CP026269.1	*K. oxytoca*	https://www.ncbi.nlm.nih.gov/nuccore/CP026269.1
CP008788.1	*K. oxytoca*	https://www.ncbi.nlm.nih.gov/nuccore/CP008788.1
CP018362.1	*K. oxytoca*	https://www.ncbi.nlm.nih.gov/nuccore/CP018362.1
CP026275.1	*K. oxytoca*	https://www.ncbi.nlm.nih.gov/nuccore/CP026275.1
CP026715.1	*K. oxytoca*	https://www.ncbi.nlm.nih.gov/nuccore/CP026715.1
CP056581.1	*K. oxytoca*	https://www.ncbi.nlm.nih.gov/nuccore/CP056581.1
CP056453.1	*K. oxytoca*	https://www.ncbi.nlm.nih.gov/nuccore/CP056453.1
CP026285.1	*K. oxytoca*	https://www.ncbi.nlm.nih.gov/nuccore/CP026285.1
CP020358.1	*K. oxytoca*	https://www.ncbi.nlm.nih.gov/nuccore/CP020358.1
CP011636.1	*K. oxytoca*	https://www.ncbi.nlm.nih.gov/nuccore/CP011636.1
CP056418.1	*K. oxytoca*	https://www.ncbi.nlm.nih.gov/nuccore/CP056418.1
CP089403.1	*K. pasteurii*	https://www.ncbi.nlm.nih.gov/nuccore/CP089403.1
JAIFQY010000015.1	*K. pasteurii*	https://www.ncbi.nlm.nih.gov/nuccore/JAIFQY010000015.1
CABGGT010000016.1	*K. pasteurii*	https://www.ncbi.nlm.nih.gov/nuccore/CABGGT010000016.1
AP023148.1	*K. pneumoniae*	https://www.ncbi.nlm.nih.gov/nuccore/AP023148.1
LR130541.1	*K. pneumoniae*	https://www.ncbi.nlm.nih.gov/nuccore/LR130541.1
CP075552.1	*K. pneumoniae*	https://www.ncbi.nlm.nih.gov/nuccore/CP075552.1
CP075275.1	*K. pneumoniae*	https://www.ncbi.nlm.nih.gov/nuccore/CP075275.1
NZ_JAAVSQ010000016.1	*K. pneumoniae*	https://www.ncbi.nlm.nih.gov/nuccore/NZ_JAAVSQ010000016.1
NZ_SOMK01000022.1	*K. pneumoniae*	https://www.ncbi.nlm.nih.gov/nuccore/NZ_SOMK01000022.1
CP044393.1	*K. pneumoniae*	https://www.ncbi.nlm.nih.gov/nuccore/CP044393.1
CP028716.1	*K. pneumoniae*	https://www.ncbi.nlm.nih.gov/nuccore/CP028716.1
FO203501.1	*K. pneumoniae*	https://www.ncbi.nlm.nih.gov/nuccore/FO203501.1
CP046939.1	*K. pneumoniae*	https://www.ncbi.nlm.nih.gov/nuccore/CP046939.1
CP040593.1	*K. pneumoniae*	https://www.ncbi.nlm.nih.gov/nuccore/CP040593.1

LR133964.1	*K. pneumoniae*	https://www.ncbi.nlm.nih.gov/nuccore/LR133964.1
CP024874.1	*K. pneumoniae*	https://www.ncbi.nlm.nih.gov/nuccore/CP024874.1
CP028176.1	*K. pneumoniae*	https://www.ncbi.nlm.nih.gov/nuccore/CP028176.1
NZ_SIZK01000003.1	*K. pneumoniae*	https://www.ncbi.nlm.nih.gov/nuccore/NZ_SIZK01000003.1
NZ_CP065833.1	*K. pneumoniae*	https://www.ncbi.nlm.nih.gov/nuccore/NZ_CP065833.1
N/A	*K. pneumoniae ATCC4352*	https://www.atcc.org/products/4352
NZ_LXMI01000022.1	*K. quasipneumoniae*	https://www.ncbi.nlm.nih.gov/nuccore/NZ_LXMI01000022.1
CP030171.1	*K. quasipneumoniae*	https://www.ncbi.nlm.nih.gov/nuccore/CP030171.1
NZ_LGAL01000005.1	*K. quasipneumoniae*	https://www.ncbi.nlm.nih.gov/nuccore/NZ_LGAL01000005.1
NZ_CP030171.1	*K. quasipneumoniae*	https://www.ncbi.nlm.nih.gov/nuccore/NZ_CP030171.1
AP022142.1	*K. quasipneumoniae*	https://www.ncbi.nlm.nih.gov/nuccore/AP022142.1
LT898450.1	*K. quasipneumoniae*	https://www.ncbi.nlm.nih.gov/nuccore/LT898450.1
CP043928.1	*K. quasipneumoniae*	https://www.ncbi.nlm.nih.gov/nuccore/CP043928.1
NZ_JAFFTJ010000001.1	*K. quasipneumoniae*	https://www.ncbi.nlm.nih.gov/nuccore/NZ_JAFFTJ010000001.1
NZ_SGRL01000050.1	*K. quasipneumoniae*	https://www.ncbi.nlm.nih.gov/nuccore/NZ_SGRL01000050.1
CP068237.1	*K. quasipneumoniae*	https://www.ncbi.nlm.nih.gov/nuccore/CP068237.1
NZ_CP068237.1	*K. quasipneumoniae*	https://www.ncbi.nlm.nih.gov/nuccore/NZ_CP068237.1
CP034136.1	*K. quasipneumoniae*	https://www.ncbi.nlm.nih.gov/nuccore/CP034136.1
CP014696.2	*K. quasipneumoniae*	https://www.ncbi.nlm.nih.gov/nuccore/CP014696.2
NZ_CP014696.2	*K. quasipneumoniae*	https://www.ncbi.nlm.nih.gov/nuccore/NZ_CP014696.2
NZ_UGLL01000001.1	*K. quasipneumoniae*	https://www.ncbi.nlm.nih.gov/nuccore/NZ_UGLL01000001.1
NZ_JVVC01000134.1	*K. quasipneumoniae*	https://www.ncbi.nlm.nih.gov/nuccore/NZ_JVVC01000134.1
NZ_JAAOBL010000006.1	*K. quasipneumoniae*	https://www.ncbi.nlm.nih.gov/nuccore/NZ_JAAOBL010000006.1
LR134196.1	*K. quasipneumoniae*	https://www.ncbi.nlm.nih.gov/nuccore/LR134196.1
CP023478.1	*K. quasipneumoniae*	https://www.ncbi.nlm.nih.gov/nuccore/CP023478.1
LR588411.1	*K. quasipneumoniae*	https://www.ncbi.nlm.nih.gov/nuccore/LR588411.1
LR134202.1	*K. quasipneumoniae*	https://www.ncbi.nlm.nih.gov/nuccore/LR134202.1
CP045641.1	*K. quasipneumoniae*	https://www.ncbi.nlm.nih.gov/nuccore/CP045641.1
CP073660.1	*K. quasipneumoniae*	https://www.ncbi.nlm.nih.gov/nuccore/CP073660.1
CAAHGF010000053.1	*K. quasipneumoniae* Kp2 CIP111839	https://www.ncbi.nlm.nih.gov/nuccore/CAAHGF010000053.1
CAAHFU010000030.1	*K. quasipneumoniae* Kp2 CIP111852	https://www.ncbi.nlm.nih.gov/nuccore/CAAHFU010000030.1
CAAHFY010000023.1	*K. quasipneumoniae* Kp4 CIP111862	https://www.ncbi.nlm.nih.gov/nuccore/CAAHFY010000023.1

CP084770.1	*K. quasipneumoniae* Kp4 CIP111869	https://www.ncbi.nlm.nih.gov/nuccore/CP084770.1
CP022823.1	*K. quasivariicola*	https://www.ncbi.nlm.nih.gov/nuccore/CP022823.1
CAAHGS010000093.1	*K. quasivariicola* CIP111871	https://www.ncbi.nlm.nih.gov/nuccore/CAAHGS010000093.1
CAAHGB010000003.1	*K. quasivariicola* Kp6 CIP111879	https://www.ncbi.nlm.nih.gov/nuccore/CAAHGB010000003.1
CABEJC010000038.1	*K. spallanzanii*	https://www.ncbi.nlm.nih.gov/nuccore/CABEJC010000038.1
CP063932.1	*K. variicola*	https://www.ncbi.nlm.nih.gov/nuccore/CP063932.1
CP010523.2	*K. variicola*	https://www.ncbi.nlm.nih.gov/nuccore/CP010523.2
CP064319.1	*K. variicola*	https://www.ncbi.nlm.nih.gov/nuccore/CP064319.1
LR607362.1	*K. variicola*	https://www.ncbi.nlm.nih.gov/nuccore/LR607362.1
CP000964.1	*K. variicola*	https://www.ncbi.nlm.nih.gov/nuccore/CP000964.1
CAAHGJ010000003.1	*K. variicola*	https://www.ncbi.nlm.nih.gov/nuccore/CAAHGJ010000003.1
CP050958.1	*K. variicola*	https://www.ncbi.nlm.nih.gov/nuccore/CP050958.1
CP017289.1	*K. variicola*	https://www.ncbi.nlm.nih.gov/nuccore/CP017289.1
CP017849.1	*K. variicola*	https://www.ncbi.nlm.nih.gov/nuccore/CP017849.1
CP065162.1	*K. variicola*	https://www.ncbi.nlm.nih.gov/nuccore/CP065162.1
NZ_SZND01000011.1	*K. variicola*	https://www.ncbi.nlm.nih.gov/nuccore/NZ_SZND01000011.1
NZ_UJZF01000014.1	*K. variicola*	https://www.ncbi.nlm.nih.gov/nuccore/NZ_UJZF01000014.1
NZ_JAFHRV010000018.1	*K. variicola*	https://www.ncbi.nlm.nih.gov/nuccore/NZ_JAFHRV010000018.1
NZ_BIHE01000005.1	*K. variicola*	https://www.ncbi.nlm.nih.gov/nuccore/NZ_BIHE01000005.1
CP063867.1	*K. variicola*	https://www.ncbi.nlm.nih.gov/nuccore/CP063867.1
NZ_LR130538.1	*K. variicola*	https://www.ncbi.nlm.nih.gov/nuccore/NZ_LR130538.1
CP013985.1	*K. variicola* Kp3.CIP108642	https://www.ncbi.nlm.nih.gov/nuccore/CP013985.1
CAAHGN010000047.1	*K. variicola* Kp5 CIP111654	https://www.ncbi.nlm.nih.gov/nuccore/CAAHGN010000047.1
CAAHGL010000054.1	*K. variicola* Kp5 CIP111870	https://www.ncbi.nlm.nih.gov/nuccore/CAAHGL010000054.1
CAAHGO010000055.1	*K. variicola* Kp5 CIP111898	https://www.ncbi.nlm.nih.gov/nuccore/CAAHGO010000055.1
CP020657.1	*Klebsiella* sp. M5al	https://www.ncbi.nlm.nih.gov/nuccore/CP020657.1
CP056483.1	*Klebsiella* sp. RHBSTW-00464	https://www.ncbi.nlm.nih.gov/nuccore/CP056483.1
CP055481.1	*Klebsiella* sp. RHBSTW-00484	https://www.ncbi.nlm.nih.gov/nuccore/CP055481.1
CABGGW010000050.1	*Klebsiella* sp. SB6422	https://www.ncbi.nlm.nih.gov/nuccore/CABGGW010000050.1
CABGGQ010000013.1	*Klebsiella* sp. SB6421	https://www.ncbi.nlm.nih.gov/nuccore/CABGGQ010000013.1
CP050321.1	*Kluyvera* gensp. 3	https://www.ncbi.nlm.nih.gov/nuccore/CP050321.1

LR134138.1	*Kluyvera intermedia*	https://www.ncbi.nlm.nih.gov/nuccore/LR134138.1
LR699009.1	*P. gergoviae*	https://www.ncbi.nlm.nih.gov/nuccore/LR699009.1
LR134195.1	*R. ornithinolytica*	https://www.ncbi.nlm.nih.gov/nuccore/LR134195.1
LR134230.1	*R. ornithinolytica*	https://www.ncbi.nlm.nih.gov/nuccore/LR134230.1
AP022438.1	*R. planticola*	https://www.ncbi.nlm.nih.gov/nuccore/AP022438.1
CP019899.1	*R. planticola*	https://www.ncbi.nlm.nih.gov/nuccore/CP019899.1
CP040183.1	*R. planticola*	https://www.ncbi.nlm.nih.gov/nuccore/CP040183.1
JMPP01000009.1	*R. planticola* CIP100751	https://www.ncbi.nlm.nih.gov/nuccore/JMPP01000009.1
LR595855.1	*R. terrigena*	https://www.ncbi.nlm.nih.gov/nuccore/LR595855.1
**Sequences used to perform *in silico* analysis for assay targeting *rapZ***		
**NCBI Accession Number**	**Species**	**URL**
NZ_JAIRBE010000019.1	*K. aerogenes*	https://www.ncbi.nlm.nih.gov/nuccore/NZ_JAIRBE010000019.1
NZ_JAFHXN010000001.1	*K. aerogenes*	https://www.ncbi.nlm.nih.gov/nuccore/NZ_JAFHXN010000001.1
CP049600.1	*K. aerogenes*	https://www.ncbi.nlm.nih.gov/nuccore/CP049600.1
CP002824.1	*K. aerogenes*	https://www.ncbi.nlm.nih.gov/nuccore/CP002824.1
NZ_CP041925.1	*K. aerogenes*	https://www.ncbi.nlm.nih.gov/nuccore/NZ_CP041925.1
N/A	*K. aerogenes* ATCC13048	https://www.atcc.org/products/13048
N/A	*K. aerogenes* ATCC49469	https://www.atcc.org/products/49469
LR134124.1	*K. aerogenes* NCTC10006	https://www.ncbi.nlm.nih.gov/nuccore/LR134124.1
LR134475.1	*K. aerogenes* NCTC9735	https://www.ncbi.nlm.nih.gov/nuccore/LR134475.1
CP059391.1	*K. africana*	https://www.ncbi.nlm.nih.gov/nuccore/CP059391.1
CP084874.1	*K. africana*	https://www.ncbi.nlm.nih.gov/nuccore/CP084874.1
CAAHGQ010000012.1	*K. africana* Kp7 CIP111653	https://www.ncbi.nlm.nih.gov/nuccore/CAAHGQ010000012.1
VNUQ01000020.1	*K. grimontii*	https://www.ncbi.nlm.nih.gov/nuccore/VNUQ01000020.1
QFVM01000025.1	*K. grimontii*	https://www.ncbi.nlm.nih.gov/nuccore/QFVM01000025.1
WMOY01000015.1	*K. grimontii*	https://www.ncbi.nlm.nih.gov/nuccore/WMOY01000015.1
VNVV01000008.1	*K. grimontii*	https://www.ncbi.nlm.nih.gov/nuccore/VNVV01000008.1
VNUP01000027.1	*K. grimontii*	https://www.ncbi.nlm.nih.gov/nuccore/VNUP01000027.1
LR594038.1	*K. grimontii* NCTC9146	https://www.ncbi.nlm.nih.gov/nuccore/LR594038.1
CP054159.1	*K. michiganensis*	https://www.ncbi.nlm.nih.gov/nuccore/CP054159.1
CP089395.1	*K. michiganensis*	https://www.ncbi.nlm.nih.gov/nuccore/CP089395.1
CP073236.1	*K. michiganensis*	https://www.ncbi.nlm.nih.gov/nuccore/CP073236.1

NZ_UGMJ01000003.1	*K. michiganensis* NCTC10261	https://www.ncbi.nlm.nih.gov/nuccore/NZ_UGMJ01000003.1
NZ_UGJV01000001.1	*K. michiganensis* NCTC8167	https://www.ncbi.nlm.nih.gov/nuccore/NZ_UGJV01000001.1
NZ_CP033844.1	*K. oxytoca*	https://www.ncbi.nlm.nih.gov/nuccore/NZ_CP033844.1
CP017928.1	*K. oxytoca*	https://www.ncbi.nlm.nih.gov/nuccore/CP017928.1
CP011618.1	*K. oxytoca*	https://www.ncbi.nlm.nih.gov/nuccore/CP011618.1
CP089399.1	*K. oxytoca*	https://www.ncbi.nlm.nih.gov/nuccore/CP089399.1
CP089411.1	*K. oxytoca*	https://www.ncbi.nlm.nih.gov/nuccore/CP089411.1
LS483483.1	*K. oxytoca* NCTC11355	https://www.ncbi.nlm.nih.gov/nuccore/LS483483.1
LR133932.1	*K. oxytoca* NCTC11356	https://www.ncbi.nlm.nih.gov/nuccore/LR133932.1
LR134333.1	*K. oxytoca* NCTC13727	https://www.ncbi.nlm.nih.gov/nuccore/LR134333.1
CABGGT010000019.1	*K. pasteurii* CIP111696	https://www.ncbi.nlm.nih.gov/nuccore/CABGGT010000019.1
CABGHD010000018.1	*K. pasteurii* SB6407	https://www.ncbi.nlm.nih.gov/nuccore/CABGHD010000018.1
CABGGP010000012.1	*K. pasteurii* SB6409	https://www.ncbi.nlm.nih.gov/nuccore/CABGGP010000012.1
CABGGO010000012.1	*K. pasteurii* SB6410	https://www.ncbi.nlm.nih.gov/nuccore/CABGGO010000012.1
CABGGV010000001.1	*K. pasteurii* SB6413	https://www.ncbi.nlm.nih.gov/nuccore/CABGGV010000001.1
CABGHE010000054.1	*K. pasteurii* SB6417	https://www.ncbi.nlm.nih.gov/nuccore/CABGHE010000054.1
CABGHB010000003.1	*K. pasteurii* SB6423	https://www.ncbi.nlm.nih.gov/nuccore/CABGHB010000003.1
CP045015.1	*K. pneumoniae*	https://www.ncbi.nlm.nih.gov/nuccore/CP045015.1
CP082290.1	*K. pneumoniae*	https://www.ncbi.nlm.nih.gov/nuccore/CP082290.1
NZ_SSYO01000004.1	*K. pneumoniae*	https://www.ncbi.nlm.nih.gov/nuccore/NZ_SSYO01000004.1
NZ_FKYN01000008.1	*K. pneumoniae*	https://www.ncbi.nlm.nih.gov/nuccore/NZ_FKYN01000008.1
NZ_FLCV01000006.1	*K. pneumoniae*	https://www.ncbi.nlm.nih.gov/nuccore/NZ_FLCV01000006.1
CDOT01000035.1	*K. pneumoniae* ATCC13884	https://www.ncbi.nlm.nih.gov/nuccore/CDOT01000035.1
ACZD01000132.1	*K. pneumoniae* ATCC13884	https://www.ncbi.nlm.nih.gov/nuccore/ACZD01000132.1
0cdd86090c324424.	*K. pneumoniae* ATCC4352	https://www.ncbi.nlm.nih.gov/nuccore/0cdd86090c324424.
CP009208.1	*K. pneumoniae* ATCC43816	https://www.ncbi.nlm.nih.gov/nuccore/CP009208.1
CP064352.1	*K. pneumoniae* ATCC43816	https://www.ncbi.nlm.nih.gov/nuccore/CP064352.1
UGMQ01000003.1	*K. pneumoniae* NCTC5046	https://www.ncbi.nlm.nih.gov/nuccore/UGMQ01000003.1

UGLV01000001.1	*K. pneumoniae* NCTC5048	https://www.ncbi.nlm.nih.gov/nuccore/UGLV01000001.1
NZ_CABDVJ010000002.1	*K. pneumoniae* NCTC9150	https://www.ncbi.nlm.nih.gov/nuccore/NZ_CABDVJ010000002.1
LR588410.1	*K. pneumoniae* NCTC9171	https://www.ncbi.nlm.nih.gov/nuccore/LR588410.1
CP084860.1	*K. pneumoniae* SB1067	https://www.ncbi.nlm.nih.gov/nuccore/CP084860.1
CP084859.1	*K. pneumoniae* SB1139	https://www.ncbi.nlm.nih.gov/nuccore/CP084859.1
CP084843.1	*K. pneumoniae* SB611	https://www.ncbi.nlm.nih.gov/nuccore/CP084843.1
CP084829.1	*K. pneumoniae* SB615	https://www.ncbi.nlm.nih.gov/nuccore/CP084829.1
NZ_CP065838.1	*K. quasipneumoniae*	https://www.ncbi.nlm.nih.gov/nuccore/NZ_CP065838.1
CP023478.1	*K. quasipneumoniae*	https://www.ncbi.nlm.nih.gov/nuccore/CP023478.1
CP078761.1	*K. quasipneumoniae*	https://www.ncbi.nlm.nih.gov/nuccore/CP078761.1
CP031257.1	*K. quasipneumoniae*	https://www.ncbi.nlm.nih.gov/nuccore/CP031257.1
CP066173.1	*K. quasipneumoniae*	https://www.ncbi.nlm.nih.gov/nuccore/CP066173.1
LR607330.1	*K. quasipneumoniae*	https://www.ncbi.nlm.nih.gov/nuccore/LR607330.1
CP082785.1	*K. quasipneumoniae*	https://www.ncbi.nlm.nih.gov/nuccore/CP082785.1
CP084818.1	*K. quasipneumoniae*	https://www.ncbi.nlm.nih.gov/nuccore/CP084818.1
LT898450.1	*K. quasipneumoniae*	https://www.ncbi.nlm.nih.gov/nuccore/LT898450.1
CP043928.1	*K. quasipneumoniae*	https://www.ncbi.nlm.nih.gov/nuccore/CP043928.1
CP026368.1	*K. quasipneumoniae*	https://www.ncbi.nlm.nih.gov/nuccore/CP026368.1
CP045641.1	*K. quasipneumoniae*	https://www.ncbi.nlm.nih.gov/nuccore/CP045641.1
CP063874.1	*K. quasipneumoniae*	https://www.ncbi.nlm.nih.gov/nuccore/CP063874.1
CP030171.1	*K. quasipneumoniae*	https://www.ncbi.nlm.nih.gov/nuccore/CP030171.1
CP029597.1	*K. quasipneumoniae* ATCC700603	https://www.ncbi.nlm.nih.gov/nuccore/CP029597.1
CP014696.2	*K. quasipneumoniae* ATCC700603	https://www.ncbi.nlm.nih.gov/nuccore/CP014696.2
CAAHGF010000089.1	*K. quasipneumoniae* Kp2 CIP111839	https://www.ncbi.nlm.nih.gov/nuccore/CAAHGF010000089.1
CAAHFU010000035.1	*K. quasipneumoniae* Kp2 CIP111852	https://www.ncbi.nlm.nih.gov/nuccore/CAAHFU010000035.1
CP034136.1	*K. quasipneumoniae* Kp4	https://www.ncbi.nlm.nih.gov/nuccore/CP034136.1
CAAHFY010000004.1	*K. quasipneumoniae* Kp4 CIP111862	https://www.ncbi.nlm.nih.gov/nuccore/CAAHFY010000004.1
CP084770.1	*K. quasipneumoniae* Kp4 CIP111869	https://www.ncbi.nlm.nih.gov/nuccore/CP084770.1
CP084803.1	*K. quasipneumoniae* Kp4 SB98	https://www.ncbi.nlm.nih.gov/nuccore/CP084803.1
LR134196.1	*K. quasipneumoniae* NCTC11357	https://www.ncbi.nlm.nih.gov/nuccore/LR134196.1
LR588411.1	*K. quasipneumoniae* NCTC9170	https://www.ncbi.nlm.nih.gov/nuccore/LR588411.1
LR134202.1	*K. quasipneumoniae* NCTC9180	https://www.ncbi.nlm.nih.gov/nuccore/LR134202.1

CAESCD010000008.1	*K. quasivariicola* Kp6	https://www.ncbi.nlm.nih.gov/nuccore/CAESCD010000008.1
VLPG01000003.1	*K. quasivariicola* Kp6	https://www.ncbi.nlm.nih.gov/nuccore/VLPG01000003.1
UJYW01000007.1	*K. quasivariicola* Kp6	https://www.ncbi.nlm.nih.gov/nuccore/UJYW01000007.1
CAAHGS010000091.1	*K. quasivariicola* Kp6 CIP111871	https://www.ncbi.nlm.nih.gov/nuccore/CAAHGS010000091.1
CAAHGB010000003.1	*K. quasivariicola* Kp6 CIP111879	https://www.ncbi.nlm.nih.gov/nuccore/CAAHGB010000003.1
CABEJC010000037.1	*K. spallanzanii* CIP111695	https://www.ncbi.nlm.nih.gov/nuccore/CABEJC010000037.1
CP050958.1	*K. variicola*	https://www.ncbi.nlm.nih.gov/nuccore/CP050958.1
LR607362.1	*K. variicola*	https://www.ncbi.nlm.nih.gov/nuccore/LR607362.1
CP047360.1	*K. variicola*	https://www.ncbi.nlm.nih.gov/nuccore/CP047360.1
CP008700.1	*K. variicola*	https://www.ncbi.nlm.nih.gov/nuccore/CP008700.1
CP001891.1	*K. variicola*	https://www.ncbi.nlm.nih.gov/nuccore/CP001891.1
CP087978.1	*K. variicola*	https://www.ncbi.nlm.nih.gov/nuccore/CP087978.1
CP087979.1	*K. variicola* Kp3	https://www.ncbi.nlm.nih.gov/nuccore/CP087979.1
CP092632.1	*K. variicola* Kp3	https://www.ncbi.nlm.nih.gov/nuccore/CP092632.1
CP088956.1	*K. variicola* Kp3	https://www.ncbi.nlm.nih.gov/nuccore/CP088956.1
CP013985.1	*K. variicola* Kp3 LMG23571	https://www.ncbi.nlm.nih.gov/nuccore/CP013985.1
CP084767.1	*K. variicola* Kp5 CDC4241-71	https://www.ncbi.nlm.nih.gov/nuccore/CP084767.1
CAAHGN010000002.1	*K. variicola* Kp5 CIP111654	https://www.ncbi.nlm.nih.gov/nuccore/CAAHGN010000002.1
CAAHGL010000004.1	*K. variicola* Kp5 CIP111870	https://www.ncbi.nlm.nih.gov/nuccore/CAAHGL010000004.1
CAAHGO010000005.1	*K. variicola* Kp5 CIP111898	https://www.ncbi.nlm.nih.gov/nuccore/CAAHGO010000005.1
CAAHGJ010000045.1	*K. variicola* Kp5 SB94	https://www.ncbi.nlm.nih.gov/nuccore/CAAHGJ010000045.1
LR134235.1	*K. variicola* NCTC9668	https://www.ncbi.nlm.nih.gov/nuccore/LR134235.1
LR588409.1	*K. variicola* NTC9178	https://www.ncbi.nlm.nih.gov/nuccore/LR588409.1

Supplementary Table S2: Sequence of synthetic double-sided DNA fragment (gBlock®) used as template for the IAC

**Sequence (5′-3′)**	AGCACCTCTAAGTAAGTGAGCGGTCGTGACATTATCCCTGATTTTCTCAC<br>TACTATTAGTACTCACGGCGCAATTCCACCACAGCCTTGTCTCGCCAGAA<br>TGCCAGTCAGCATAAGGAAGAGCTCAAGGCAGGTCAACTCGCACTGTGAG<br>GGTCACATGGGCGTTCGGCACTACCGACACGAACCTCAGTTAGCGTACAT<br>CCTACCAGAGGTCTGTGGCCCCGTGGTCAAAAGTGCGGGTTTCGTATTTG<br>CTGCTCGTCAGTACTTTCAGAATCATGACCTGCACGGCAAAGAGACGCTT<br>ATTATGGAGCTCGACATGGCAATAACGCGACGAATCTACGTCACGACGAG<br>AATAGTGTAAACGAAGCTGCTGACGGCGGAAGCGTCAAAGGGGTCTGTGA<br>ATTGTTATTCGCGAAAAACATCCGTCCCCGTGGGGGATAGTCACCGACGC<br>CGTTTTATAGAAGCCTAGGGGAACAGGTTGGTTTAACTAGCTTAAGAA

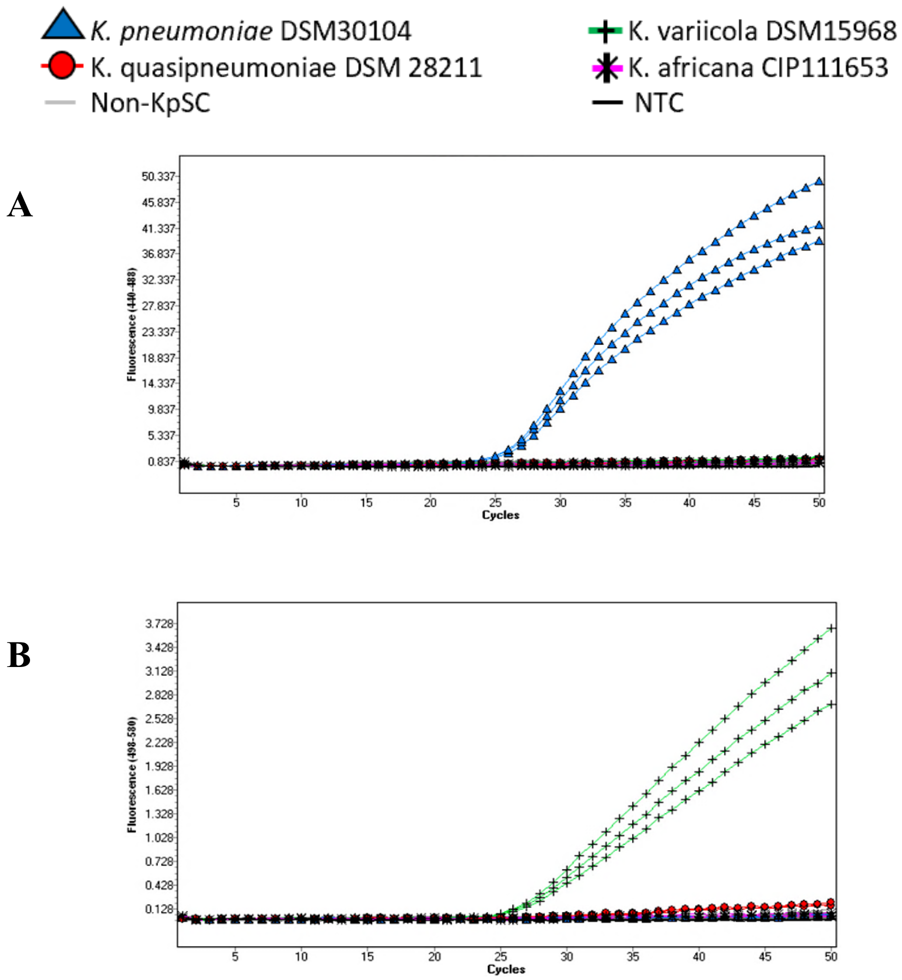

C

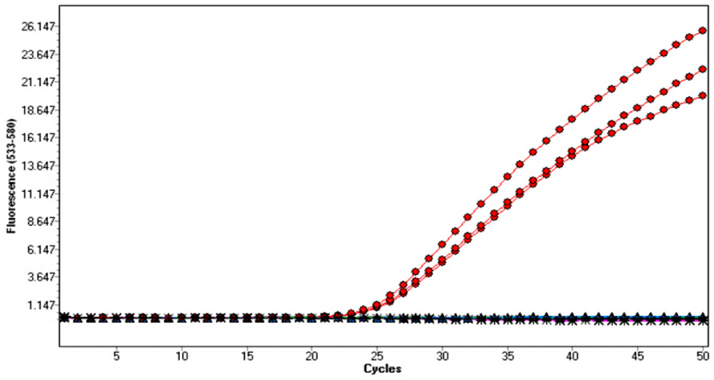

D

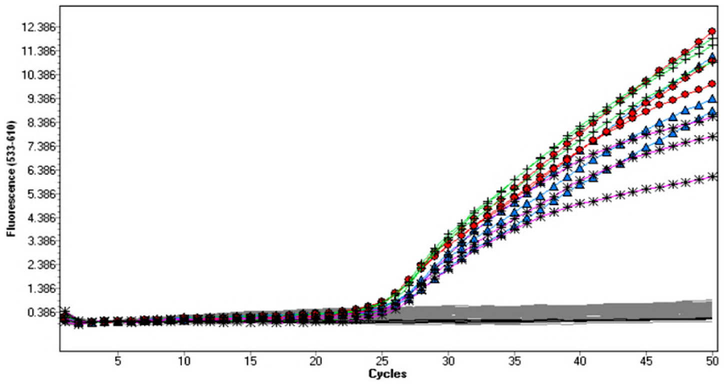

Supplementary Figure S1: PCR amplification curves produced during exclusivity testing for KpSC multiplex using 10,000 GE of each species as template

Fig S3.1 (A) Amplification curves for *K. pneumoniae* DSM30104 (triangles) using the *lepA* assay in the CYAN500 channel (440-448 nm); (B) Amplification curves for *K. variicola* DSM15968 (circles) using the *lepA* assay in the FAM channel (498-580 nm); (C) Amplification curves for *K. quasipneumoniae* DSM28211 (crosses) using the *rapZ* assay in the HEX channel (533-580 nm); (D) Amplification curves for KpSC members (*K. pneumoniae* DSM30104 (triangles) *K. variicola* DSM15968 (circles) *K. quasipneumoniae* DSM28211 (crosses) and *K. africana* CIP111653 (stars) using the ZKIR assay in the ROX channel (533-610 nm).

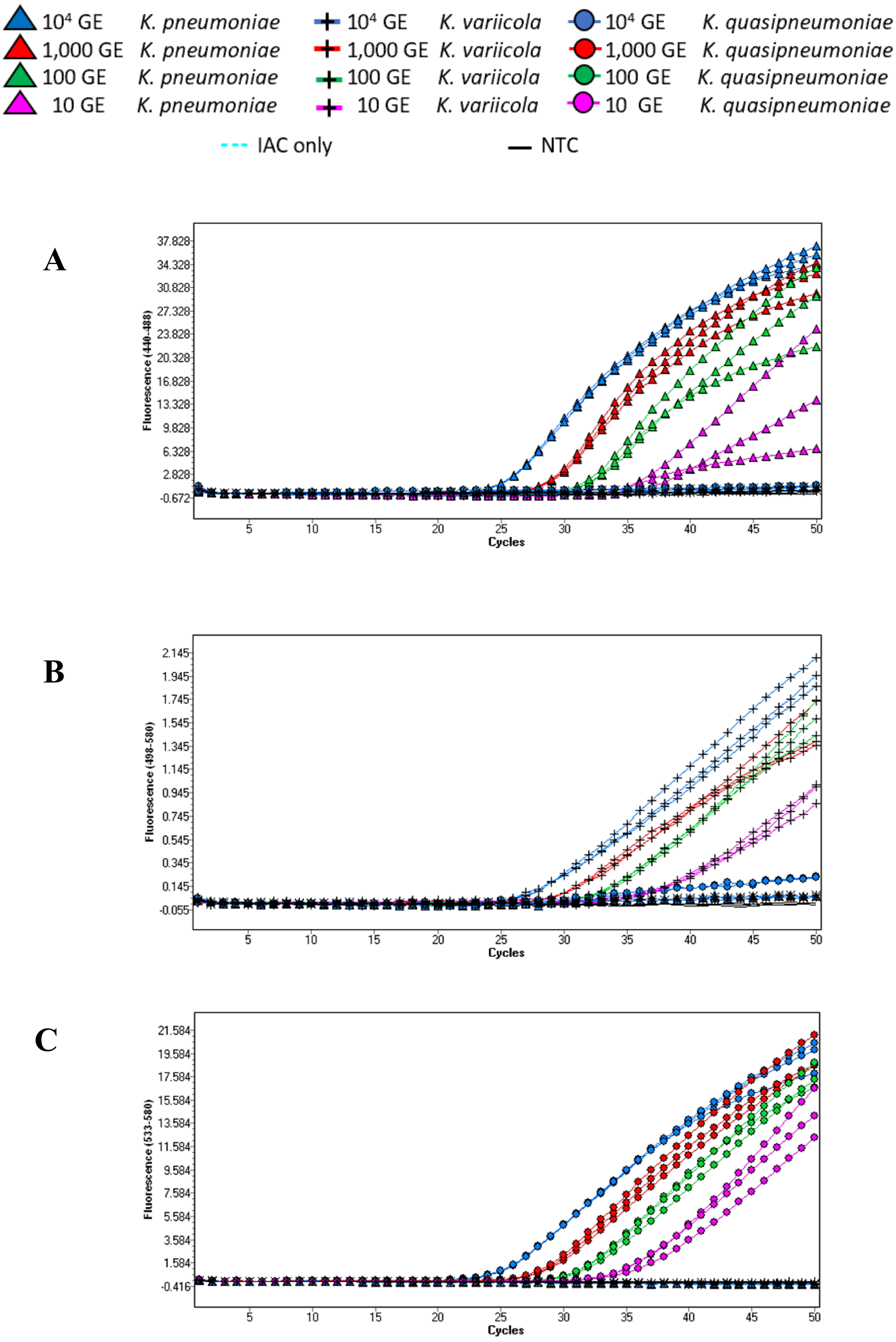

10⁴ GE K. pneumoniae
1,000 GE K. pneumoniae
100 GE K. pneumoniae
10 GE K. pneumoniae
10⁴ GE K. variicola
1,000 GE K. variicola
100 GE K. variicola
10 GE K. variicola
10⁴ GE K. quasipneumoniae
1,000 GE K. quasipneumoniae
100 GE K. quasipneumoniae
10 GE K. quasipneumoniae
IAC only
NTC
A
Fluorescence (440-488)
37.828
34.328
30.828
27.328
23.828
20.328
16.828
13.328
9.828
6.328
2.828
-0.672
5
10
15
20
25
30
35
40
45
50
Cycles
B
Fluorescence (498-580)
2.145
1.945
1.745
1.545
1.345
1.145
0.945
0.745
0.545
0.345
0.145
-0.055
5
10
15
20
25
30
35
40
45
50
Cycles
C
Fluorescence (533-580)
21.584
19.584
17.584
15.584
13.584
11.584
9.584
7.584
5.584
3.584
1.584
-0.416
5
10
15
20
25
30
35
40
45
50
Cycles

D

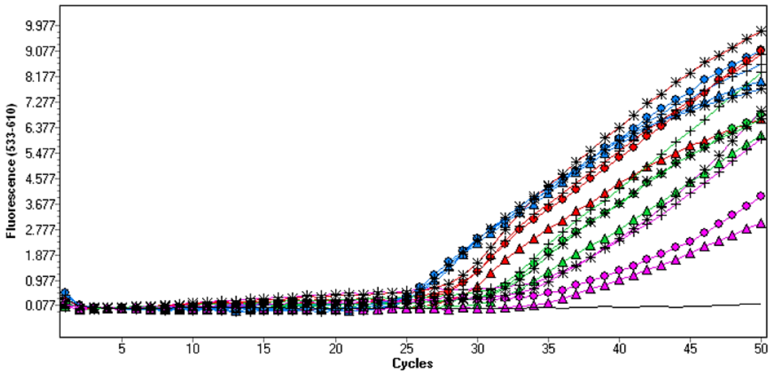

E

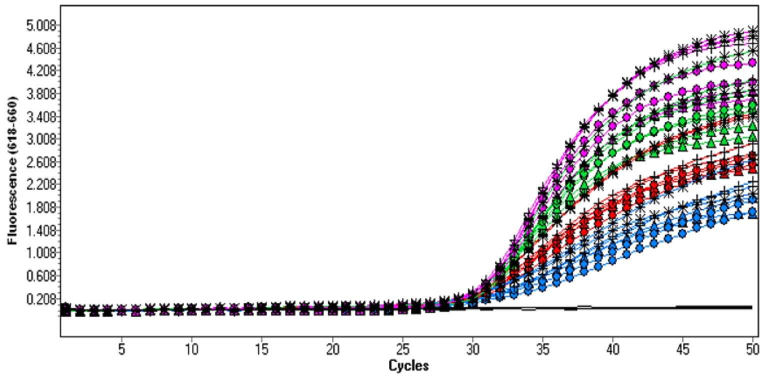

Supplementary Figure S2: PCR amplification curves demonstrating detection of target species to 10 GE

Figure S2 (A) Amplification curves for *K. pneumoniae* DSM30104 dilution series (triangles) using the *lepA* assay in the CYAN500 channel (440-448 nm); (B) Amplification curves for *K. variicola* DSM15968 (circles) using the *lepA* assay in the FAM channel (498-580 nm); (C) Amplification curves for *K. quasipneumoniae* DSM28211 (crosses) dilution series using the *rapZ* assay in the HEX channel (533-580 nm). Amplification curves for a dilution series of all KpSC members (*K. pneumoniae* DSM30104 (triangles) *K. variicola* DSM15968 (circles) *K. quasipneumoniae* DSM28211 (crosses) using the ZKIR assay in the ROX channel (533-610 nm) with all non-KpSC members labelled with grey lines; (E) Amplification curves for IAC assay targeting the synthetic construct with the no template control visible as a solid black line in the Cy5 channel (618-660 nm). For all assays (A-D) 10,000 GE = blue; 1,000 GE = red; 100 GE = green; 10 GE = pink
